# A Generally Applicable Method for Disentangling the Effect of Individual Noncovalent Interactions on the Binding Energy

**DOI:** 10.1002/anie.202421922

**Published:** 2024-12-11

**Authors:** Ahmet Altun, Isaac F. Leach, Frank Neese, Giovanni Bistoni

**Affiliations:** ^1^ Max-Planck-Institut für Kohlenforschung, Kaiser-Wilhelm-Platz 1 45470 Mülheim an der Ruhr Germany; ^2^ Department of Chemistry, Biology and Biotechnology University of Perugia 06123 Perugia Italy

**Keywords:** Local Energy Decomposition, noncovalent interactions, interaction energy, correlation energy, London Dispersion, DLPNO-CCSD(T), HFLD

## Abstract

We introduce the fragment‐pairwise Local Energy Decomposition (fp‐LED) scheme for precise quantification of individual interactions contributing to the binding energy of arbitrary chemical entities, such as protein‐ligand binding energies, lattice energies of molecular crystals, or association energies of large biomolecular assemblies. Using fp‐LED, we can assess whether the contribution to the binding energy arising from noncovalent interactions between pairs of molecular fragments in any chemical system is attractive or repulsive, and accurately quantify its magnitude at the coupled cluster level ‐ commonly considered as the “gold standard” of computational chemistry. Such insights are crucial for advancing molecular and material design strategies in fields like catalysis and therapeutic development. Illustrative applications across diverse fields demonstrate the versatility and accuracy of this theoretical framework, promising profound implications for fundamental understanding and practical applications.

## Graphical abstract

A fragment‐pairwise Local Energy Decomposition (fp‐LED) Scheme is introduced for precise quantification of individual interactions within complex molecular systems. Applications across diverse fields, encompassing protein‐ligand binding energies and association energies in large biomolecular assemblies, showcase the versatility and broad applicability of this approach.

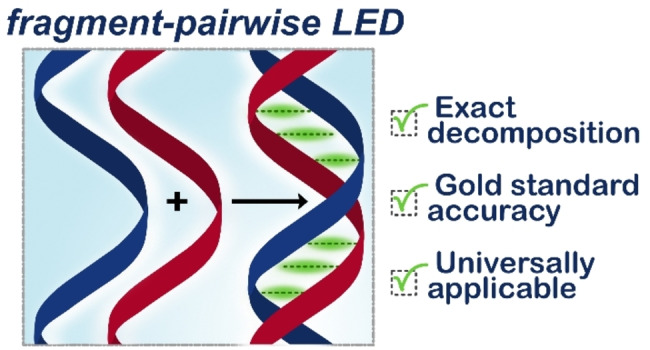

## Introduction

Quantifying individual interactions within complex molecular systems, such as proteins, enzymes, DNA, RNA, crystals, surfaces, and self‐assembled/aggregated systems in solid and solution phases is pivotal for understanding the origin of their intricate structure, function, reactivity, dynamics, and properties, thus catalyzing advancements across myriad fields.[^1^, ^2^] Such understanding underpins the exploitation of processes governing catalysis, signal transduction, and gene regulation, eventually aiding the discovery of novel therapeutic targets and safer drugs with optimal binding affinity to target biomolecules through specific pathways.^[3]^ Moreover, quantifying interactions between catalysts and reactants unveils invaluable insights into reaction pathways and kinetics, driving the fine‐tuning of catalytic processes for industrial applications such as chemical or fuel production.^[4]^ In materials science, where intermolecular interactions govern material properties like strength, conductivity, and optical characteristics, their accurate quantification is paramount for engineering materials tailored for diverse applications in electronics, aerospace, healthcare, and beyond.^[5]^ Additionally, understanding interactions between early biomolecules, including nucleic acids and amino acids, serves as a linchpin in elucidating the origins of life.^[6]^

From a fundamental point of view, a chemical interaction, whether covalent or noncovalent, intermolecular or intramolecular, refers to the influence that arbitrarily defined subsystems (or fragments) within a complex chemical system exert on each other. Computational methods are fundamentally important for their identification and quantification, offering insights that are often unattainable through experimental means alone. For systems with just two fragments, the effect of chemical interactions on the energy (denoted hereafter as “interaction energy”) can often be estimated computationally by calculating the energy difference between two points on the potential energy surface of the system. By contrast, for complex chemical systems with multiple interacting units, disentangling the contributions from different chemical interactions can be extremely challenging. An ideal computational methodology should provide an accurate and efficient quantification of all fragment‐pairwise interactions across the entire system, regardless of fragment definitions. This means the method should be versatile and robust enough to handle different ways of dividing the system into fragments, ensuring that the results are consistent and reliable. Importantly, the interaction strength of a given fragment pair can be significantly influenced by the rest of the system. Therefore, it is crucial that the chosen computational methodology accurately incorporates these “environmental” effects.

A plethora of quantum chemical methods has been developed over the years for the study of chemical interactions, each one with its own set of assumptions and limitations. Symmetry Adapted Perturbation Theory (SAPT)[^7^, ^8^, ^9^, ^10^, ^11^] has proven extremely successful for the study of weakly interacting dimers. Extending this approach beyond dimers to study systems with many interacting fragments is a focus of intense research efforts.[^12^, ^13^, ^14^, ^15^, ^16^, ^17^, ^18^, ^19^, ^20^, ^21^] Orbital‐space‐based Energy Decomposition Analysis (EDA) Schemes,^[22],[23]^ which are mostly based on the pioneering work of Morokuma,^[24],[25]^ are also useful tools for the study of chemical interactions. However, their application is also limited to isolated fragment pairs. Real space quantification methods, typically based on the Quantum Theory of Atoms in Molecule (QTAIM),[^26^, ^27^, ^28^] offer a promising avenue for unraveling the contribution from individual molecular interactions in complex systems, allowing for arbitrary fragment definitions. Unfortunately, the quantification of interaction energies within these approaches requires up to six‐dimensional numerical integrations over the irregularly‐shaped atomic basins, limiting the efficiency[^29^, ^30^, ^31^] and accuracy[^31^, ^32^] of the approach. Addressing these limitations remains a focus for advancing real space energy decomposition schemes.[^33^, ^34^, ^35^, ^36^, ^37^] An alternative strategy to study chemical interactions in complex systems relies on fragmentation methods[^38^, ^39^, ^40^] in which the total energy of a system is expressed as a sum of contributions from all possible n‐tuples of fragments, *e.g*., monomers, dimers, trimers and so on. As this expansion needs to be truncated in practical applications, the accuracy of such computations is strongly affected by the choice of the computational strategy used for incorporating environmental effects. Hence, a generally applicable quantum chemical method that is capable of providing a quantitative measure of the strength of individual chemical interactions to the energy of a complex chemical system is still lacking.

Our strategy to address this problem relies on the well‐established Local Energy Decomposition (LED)[^41^, ^42^, ^43^, ^44^] scheme at the domain‐based local pair natural orbital cluster level incorporating singles, doubles, and perturbative triples, *i.e*., DLPNO‐CCSD(T).[^45^, ^46^, ^47^, ^48^, ^49^, ^50^, ^51^, ^52^, ^53^, ^54^] Additionally, we employ the cost‐effective Hartree‐Fock plus London Dispersion (HFLD)^[55],[56]^ variant, which is specifically designed to enable efficient analysis of noncovalent interactions. DLPNO‐CCSD(T) can be applied to very large molecular systems, such as entire proteins,^[53]^ with the largest studied system to date including ~21000 basis functions and ~2100 correlated electrons.^[53]^ The HFLD scheme, capable of handling systems of similar size, offers a substantial efficiency gain over the parent DLPNO‐CCSD(T) methodology^[57]^ by introducing a series of physically motivated approximations.

For a complex system with an arbitrary number of fragments, the LED scheme allows the *exact* decomposition of the electronic energy into additive terms, corresponding to the fragment energies (also called “intra‐fragment” terms) and their pairwise interactions. The latter can also be decomposed into contributions from fragment‐pairwise electrostatics, exchange and London dispersion (LD), thus providing additional physical insight. This approach has proven to be instrumental in both the strong‐ and weak‐interaction regimes for closed‐ and open‐shell systems, and thus finds widespread application.[^41^, ^42^, ^43^, ^58^, ^59^, ^60^, ^61^, ^62^, ^63^, ^64^] LED also provides a simple definition for cooperativity effects and many‐body terms.^[63]^

A primary application area for the LED scheme lies in dissecting binding energies between two or more complex chemical entities, each one potentially constituting of many fragments, into contributions from individual fragment‐pairwise interactions. This is highly relevant in fields such as supramolecular chemistry, catalysis, materials science and drug discovery. For instance, the LED scheme can provide insights into the contribution of individual ligand‐residue interactions to the binding energy of a ligand to a protein receptor.^[62]^ However, when more than two fragments are considered, it cannot be used to quantify the net effect of the interaction between two fragments (*e.g*., a residue in the active site of the protein and a functional group in the ligand) on the binding energy of two chemical entities (*e.g*., a protein and a ligand). In this study, we will extend the LED scheme to quantify all “fragment‐pairwise” (fp) interaction energies. This scheme, denoted hereafter as fp‐LED, will enable us to ascertain whether interactions between molecular fragment pairs in any chemical system are overall attractive or repulsive, and to precisely measure their strength at the coupled cluster level. This would significantly improve our capacity to predict molecular behavior and properties, facilitating advancements across various fields of chemical research.

The fp‐LED scheme requires only a single supramolecular DLPNO‐CCSD(T) calculation. We illustrate this method with examples from various fields of chemical research, addressing a range of representative problems:

- Determining the individual interaction energies that contribute to the association energy of molecular clusters (Figure 1a
  **Figure 1:**
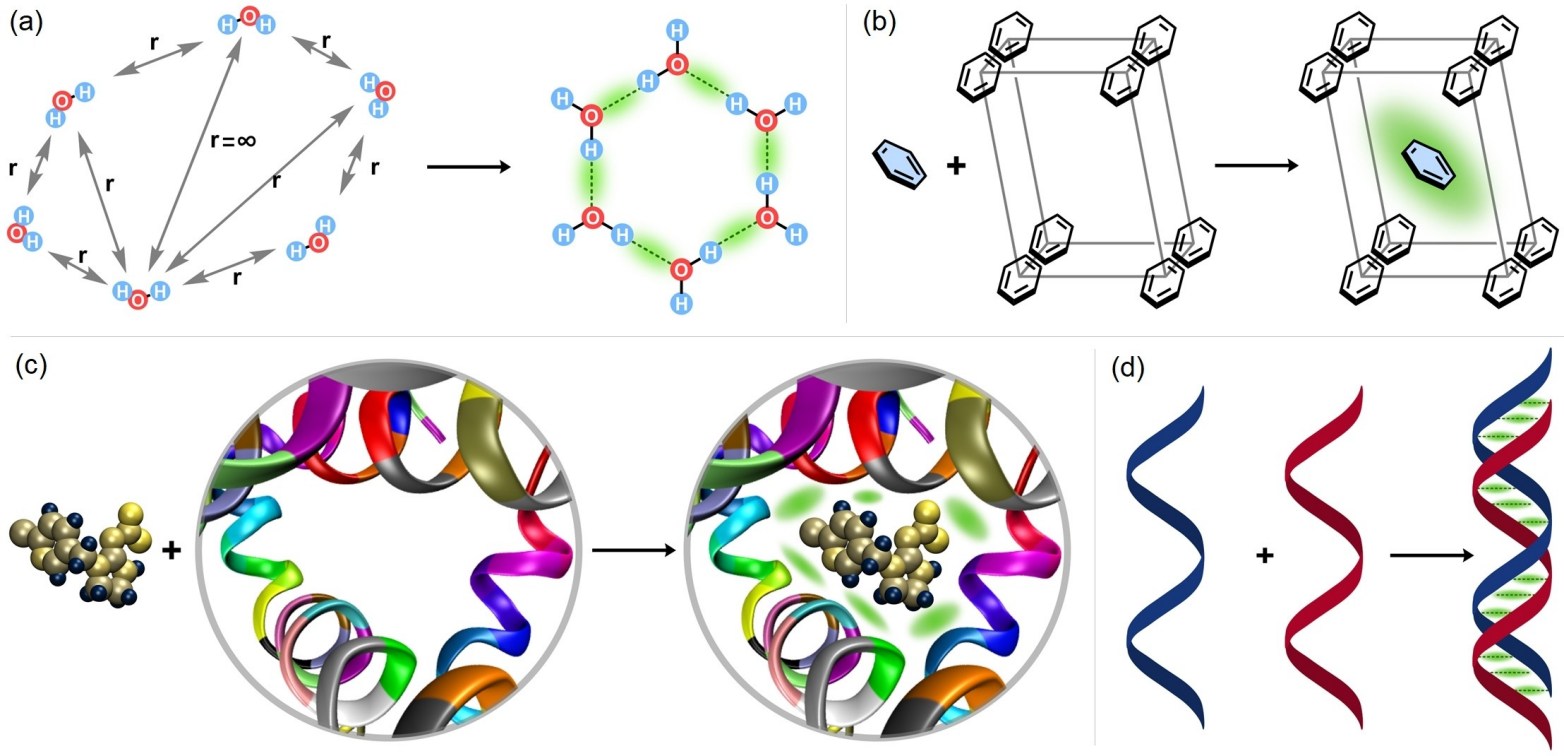
Schematic examples of inter‐fragment interactions responsible for the binding strength of different chemical entities for (a) small molecules in a molecular cluster, (b) a molecule and its neighbors in a molecular crystal, (c) a protein and a ligand, (d) two strands in the DNA duplex. The key inter‐fragment interactions that are formed upon the binding process are emphasized in green.
  );
- Unraveling the contributions of individual interactions to the lattice energies of molecular solids (Figure 1b **)**;
- Decomposing protein‐ligand interactions to reveal the contributions from individual ligand‐residue and residue‐residue interactions (Figure 1c);
- Quantifying the contributions of base‐pairing and stacking interactions to the stability of DNA duplexes (Figure 1d).

In all cases, the proposed method also allows for a clear‐cut quantification of the role played by cooperativity effects for different chemical interactions, thus offering additional physical insights.

## Results and Discussion

### Fragment‐Pairwise Local Energy Decomposition: The Water Trimer as an Illustrative Example

An in depth discussion of the theory underlying the LED scheme can be found elsewhere.[^41^, ^44^, ^55^, ^56^, ^57^, ^58^, ^61^, ^63^, ^64^] All quantum chemical calculations were carried out with a development version of the ORCA program package based on version 5.0.[^65^, ^66^, ^67^, ^68^] LED maps were generated from the ORCA output files using the LED Analysis Wizard (LEDAW) tool,^[69]^ a program suite designed to facilitate the extraction of data from LED outputs in ORCA, enabling the creation of both standard and fragment pair (fp‐LED) interaction energy maps.

**Figure 2:**
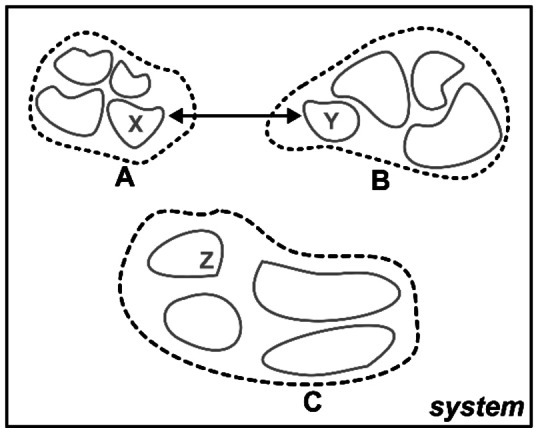
Schematic representation of a supramolecular complex ABC, composed of three predefined chemical subsystems (A, B, and C). The pairwise interaction between the X and Y fragments (located within the subsystems A and B, respectively) is indicated with an arrow. LED enables the decomposition of the total energy into interaction terms among multiple predefined subsystems. Each subsystem may consist of a single fragment or, optionally, multiple fragments to allow for the decomposition of pairwise interactions between fragments across different subsystems. Fragments may consist of single atoms or groups of atoms.

In this section, we describe how LED can be used to obtain an *exact* decomposition of the DLPNO‐CCSD(T) binding energy of M subsystems (with labels *A, B, C*, …) in a supramolecular complex into contributions originating from the interaction of pairs of fragments (with labels *X, Y, Z*, …), as shown schematically in Figure 2. Each subsystem is divided into several fragments (denoted *N*_A_, *N_B_*, *N_C_*, etc. for subsystem *A, B, C*, …, respectively), resulting in a total of N fragments. Note that *N*_A_ ≥ 1 and hence *N*_A_≥*M*. Unlike perturbative methods, which approximate total energy by summing the energies of isolated subsystems and their interaction terms, LED decomposes the total energy directly into its constituent subsystem/fragment energies.

The DLPNO‐CCSD(T) binding energy between *M* interacting subsystems can be computed using a supramolecular approach as:
(1)

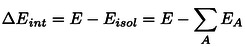




where E is the total electronic energy of the system, $E_{A}$ is the electronic energy of subsystem A, and $E_{isol}$ is the sum of the energy of the isolated noninteracting subsystems. The geometric preparation energy term (also called “strain energy”) and the thermodynamic corrections to the Gibbs free energy can also be computed separately and added to ${\Delta E}_{int}$ when needed.

For the supramolecular complex with *M* interacting subsystems and *N* fragments, the LED scheme allows the extraction of all intra‐fragment *E* ^*(X)*^ and inter‐fragment *E* ^*(,X,Y)*^ contributions to the electronic energy *E*: 
(2)

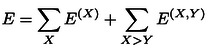




The inter‐fragment terms $E^{\left(X,Y\right)}\;$ can be repulsive (positive) or attractive (negative) and can be further decomposed into various physical components. These include the electrostatic interaction between the fragments (*E_elstat_*), the Slater exchange (*E_exch_*), the LD energy (*E_disp_*), and the nondispersive fragment‐pairwise correlation contribution (*E*_*no‐disp*_).

As each fragment can be assigned to a subsystem, it is now useful to rewrite equation 2 as:
(3)

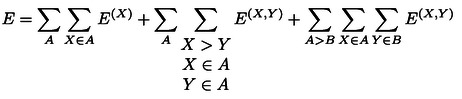




In this way, the second term incorporates all inter‐fragment contributions for fragments within the same subsystem, while the third term includes the contribution from pairs of fragments located on different subsystems. A similar equation can be written for $E_{isol}$ :
(4)

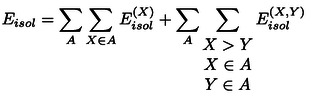




In this case, the term representing fragment‐pairwise interactions between different subsystems is missing. Inserting equations 3 and 4 into equation 1 one obtains the following expression for the interaction energy:
(5)

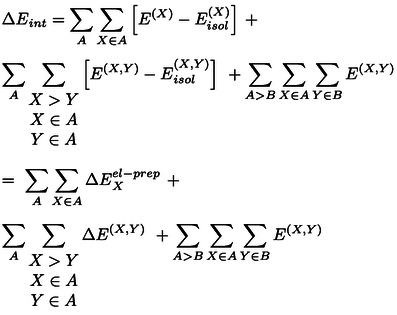




where the electronic preparation term ${\Delta E}_{X}^{el-prep}$ is the change in the energy of a fragment upon the interaction of the subsystems in the supramolecular complex; 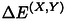 represents the corresponding change in the fragment‐pairwise interactions for fragments within the same subsystem, and the last term in the equation encompasses fragment‐pairwise interactions between different subsystems. Removing the sum over the subsystems and extending the sum over all fragments irrespective of their location, we can now write:
(6)

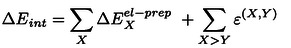




in which 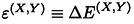 when *X* and *Y* are located on the same subsystem, and 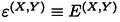 otherwise. Importantly, the only term in the sum that is not pairwise additive is ${\Delta E}_{X}^{el-prep}$ , which incorporates the dominant repulsive contributions for the interaction of *X* with the remaining *N*–1 fragments. This includes contributions from the interaction of a fragment with all other fragments. Disentangling the contributions from pairs of fragments within ${\Delta E}_{X}^{el-prep}\;$ would open up unprecedented opportunities for the accurate quantification of interaction energies in complex systems.

Since ${\Delta E}_{X}^{el-prep}$ arises from the perturbation of the electronic structure of individual fragments upon binding, the greater the inter‐fragment interaction, the larger the resulting perturbation (${\Delta E}_{X}^{el-prep}$ ). To demonstrate this, on two‐fragment *XY* systems, *i.e*., on the adducts in the S66 benchmark set^[70]^ for noncovalent interactions and on potential energy surfaces of both weakly interacting Ar_2_ and strongly interacting dihydroimidazole⋅⋅⋅BF_3_ Lewis pair, including the repulsive interaction regime, we investigated the correlation between ${\Delta E}_{X}^{el-prep}+{\Delta E}_{Y}^{el-prep}$ and $E^{\left(X,Y\right)}$ and found strong linear correlations with *R* ^*2*^=0.999, 0.996, and 0.997, respectively (see Figures S1 and S2).

This linear correlation can be exploited to quantify the electronic preparation energy contribution to the interaction of a given fragment pair *XY* in the supramolecular complex, ${\Delta E}_{XY}^{el-prep}$ . It can be obtained from ${\Delta E}_{X}^{el-prep}$ and ${\Delta E}_{Y}^{el-prep}$ as:
(7)

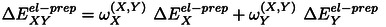




where
(8)

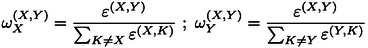




We note that a conceptually similar expression was previously used at the MP2 level using Interacting Quantum Atoms (IQA) decomposition analyses.^[71]^ The normalization factors in the denominators ensure that the sum of non‐distributed electronic preparation energies is equal to the sum of the pairwise distributed electronic preparation energies.

By summing up the electronic preparation energy contribution to the interaction of a given fragment pair ${\Delta E}_{XY}^{el-prep}$ with the corresponding fragment‐pairwise interaction term $\epsilon^{\left(X,Y\right)},$ one can quantify the overall contribution to the binding energy between the subsystems originating from the interaction between the two fragments, 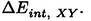 This allows us to write the binding energy as a sum of fragment‐pairwise contributions:
(9)

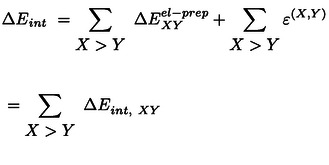




**Figure 3:**
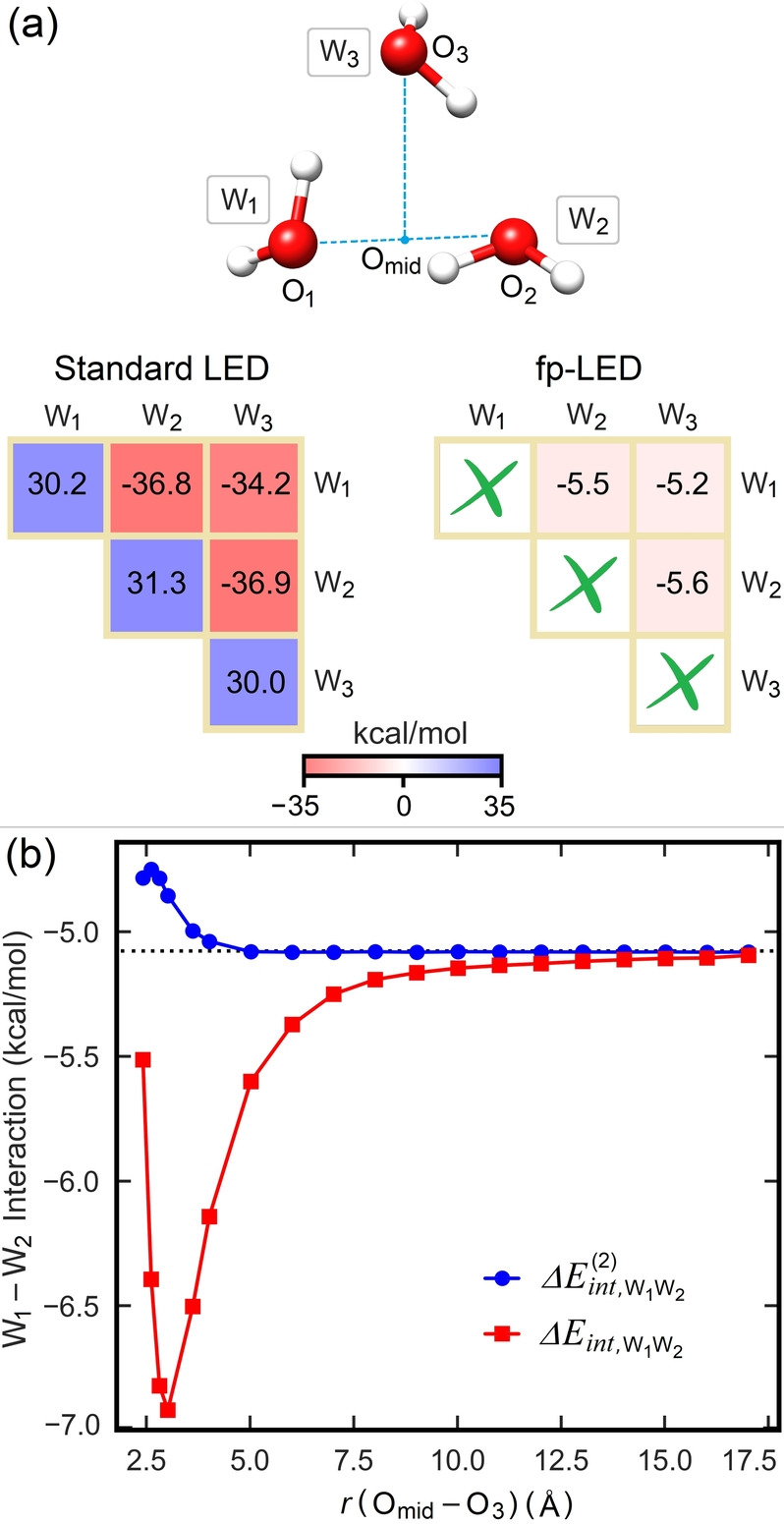
On the cyclic water trimer, (a) comparison of DLPNO‐CCSD(T_1_)/CPS(6/7)/CBS(3/4)‐level standard and fragment‐pairwise LED interaction energy maps at the equilibrium geometry (b) the dependence of the interaction energy between **W_1_** and **W_2_** (
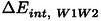
 ) on the distance with the third water molecule **W_3_** (red line), extracted using the fragment pairwise LED scheme. The corresponding two‐body term at the same geometry (${\Delta E}_{int,\;W1W2}^{\left(2\right)}$ ) is also shown for comparison (blue line).

Equation 9 represents the core idea behind the fp‐LED scheme presented here, providing an exact decomposition of binding energy between subsystems computed at the coupled cluster level into fragment‐pairwise interaction contributions. By “*exact* decomposition”, we mean that the sum of decomposed terms reconstructs ${\Delta E}_{int}$ exactly. As seen in Figure 3a on water trimer, while the standard LED interaction energy map contains very large positive and negative values, fp‐LED directly provides the individual interaction energy strengths, which are comparable to those of isolated dimers but at the same time inherently include the effect of chemical environment, a phenomenon known as “cooperativity”.

The relationship between the fragment‐pairwise decomposition obtained with the fp‐LED scheme and those derived from perturbative and/or many‐body expansion (MBE) methods merits further discussion. In MBE and perturbative schemes, the approach begins with isolated fragments, incrementally adding two‐ and higher‐order interaction terms to approximate *ΔE_int_* as closely as possible, typically truncating the expansion at the two‐ or three‐body level. In contrast, fp‐LED starts with the exact *ΔE_int_* computed using a supramolecular approach, and performs its exact decomposition into “effective” two‐body contributions. Hence, these LED fragment‐pairwise terms inherently include environmental effects, capturing many‐body contributions.

Consequently, the fp‐LED scheme also provides a quantitative framework for analyzing “cooperativity effects.” For a given fragment pair, the cooperativity contribution to the interaction can be quantified by subtracting the corresponding two‐body term (in the standard MBE) from the respective fp‐LED term. Repeating this procedure for all fragment pairs allows us to obtain the cooperativity matrix (see Supporting Information for details).

As an illustrative example, we show in Figure 3b how the binding energy 
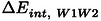
 of two water molecules **W_1_** and **W_2_** is influenced by the interaction of the third water molecule **W_3_** due to cooperative effects.

When **W_3_** is placed at a large distance of separation, the interaction energy of the water dimer at the DLPNO‐CCSD(T) level is −5.1 kcal/mol, which is consistent with state‐of‐the‐art reference values (see Table S2). As **W_3_** approaches, 
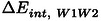
 significantly decreases due to cooperativity effects between **W_1_**–**W_2,_ W_1_**–**W_3_** and **W_2_**–**W_3_** interactions (red line in Figure 3b), reaching up to −6.9 kcal/mol. At the same time, the two‐body contribution to the binding energy ${\Delta E}_{int,W1W2\;}^{\left(2\right)}\;$ of the dimer frozen at the trimer geometry increases (blue). The difference between 
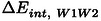
 and ${\Delta E}_{int,W1W2\;}^{\left(2\right)}\;$ represents the net contribution of cooperative effects to the **W_1_**–**W_2_** interaction. The increase in the water dimer binding strength due to cooperative effects can be explained by the increase in the dipole moment of **W_1_** and **W_2_** upon the interaction with **W_3_**. Our approach allows us to quantify this effect quantitatively at various distances of separation, thus providing a simple theoretical framework in which to analyze cooperativity of intermolecular interactions in complex systems.

### Breakdown of Binding Energies in Molecular Clusters: The Water Hexamer

**Figure 4:**
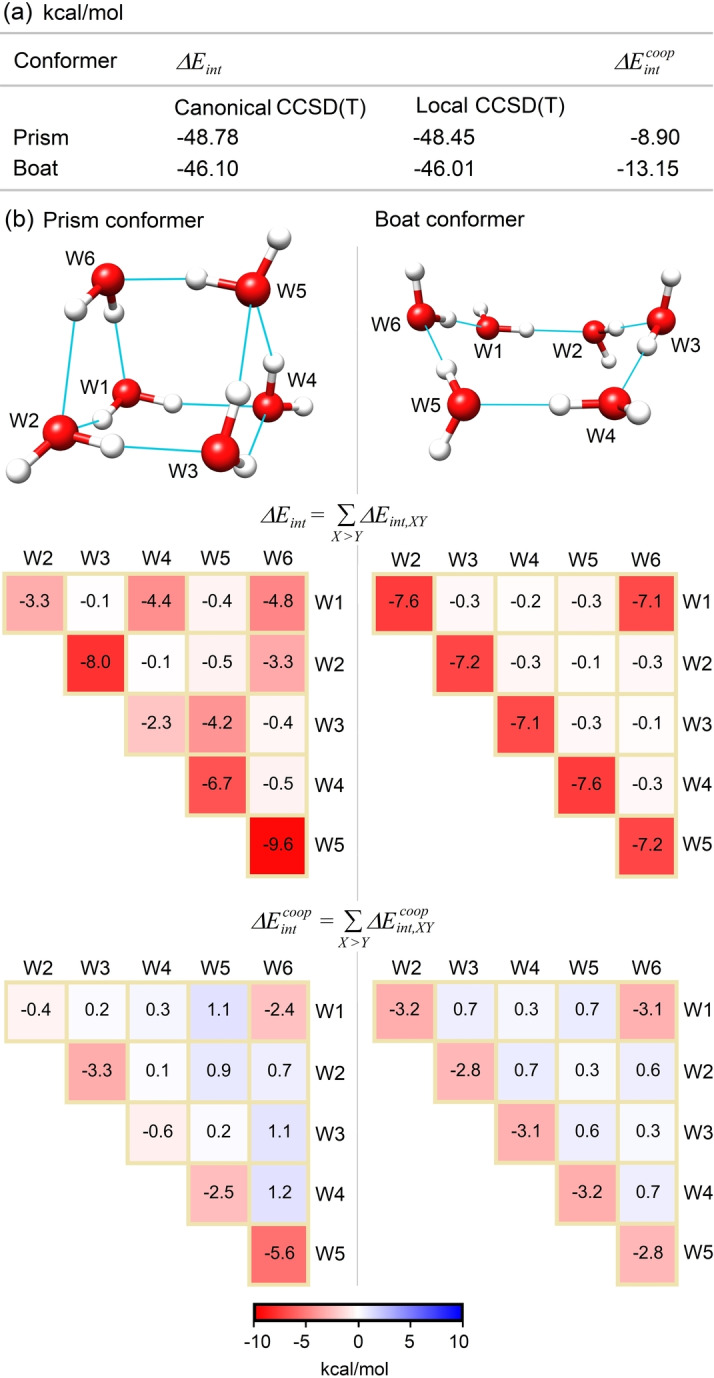
(a) Binding energies of two conformers of the water hexamer at the canonical CCSD(T)/CBS(3/4) and (local) DLPNO‐CCSD(T_1_)/CPS(6/7)/CBS(3/4) levels together with their cooperative component. (b) Fragment‐pairwise LED interaction maps providing a decomposition of water hexamer binding energies and their cooperative components into fragment‐pairwise contributions.

As a first case study, we discuss how fragment‐pairwise LED can be used to unravel the intricate pattern of noncovalent interactions responsible for the stability of small molecular clusters. Specifically, we investigated the interactions contributing to the stability of two different conformers of the water hexamer, namely the prism and boat conformers. The binding energy $\Delta E_{int}\;$ of both conformers with respect to the dissociation limit is shown in Figure 4a using CCSD(T) in its canonical and local (DLPNO) variants, with two‐point extrapolation to the basis set and PNO limits (see Supporting Information for computational details). The comparison of the results obtained with the two methods demonstrates that there is no significant error associated with the local approximation in this case. The cooperativity contribution to the binding energy, $\Delta E_{int}^{coop}\;$ is also reported for both systems at the DLPNO‐CCSD(T) level. The structures of both conformers are shown in Figure 4b, together with the fragment‐pairwise decomposition of $\Delta E_{int}$ and $\Delta E_{int}^{coop}\;$ at the DLPNO‐CCSD(T)/LED level

The LED breakdown of $\Delta E_{int}$ allows for a clear‐cut quantification of all hydrogen‐bonding interactions in these systems. These interactions are visualized in Figure 4b using the “fragment‐pairwise LED interaction maps”, where each element in the map corresponds to a specific interaction between two fragments in the molecular cluster and the entire interaction energy – including the electronic preparation – is distributed among the fragment pairs. The water molecules in the boat conformer are engaged in hydrogen bonds of similar strength, with interaction energies ranging from –7.1 to −7.6 kcal/mol. By contrast, the hydrogen bonding interaction energies in the prism conformer vary from –2.3 to −9.8 kcal/mol. The origin of this difference lies in the hydrogen bonding patterns of the two conformers. In the boat conformer, each water molecule donates one proton to another water molecule while simultaneously accepting one proton from a different water molecule. In contrast, the prism conformer features water molecules that accept or donate varying numbers of hydrogen bonds. Generally, the strongest interactions occur when a water molecule with both lone pairs involved in hydrogen bonds donates one of its protons to a water molecule with both protons engaged in hydrogen bonds, as seen in the **W_2_**–**W_3_** and **W_5_**–**W_6_** interactions in the prism conformer. This effect is inherently cooperative, arising from the charge redistribution: the hydrogen bond donor atom becomes more negatively charged upon the formation of a hydrogen bond, while the acceptor atom becomes more positively charged. Indeed, decomposition of $\Delta E_{coop}\;$ into fragment‐pairwise terms quantifies these chemically intuitive findings, showing larger values precisely for the **W_2_**–**W_3_** and **W_5_**–**W_6_** interactions in the prism conformer.

These results show that our approach allows for a precise quantification of the interaction energy between subunits in a molecular cluster. The scheme is generally valid irrespective of the system nature and provides unambiguous quantitative results, offering insights that go beyond those obtained from simple distance‐based analyses. Unlike such distance‐based analyses, LED can be used to elucidate the relative strength of different interaction types (such as H‐bond *vs*. halogen‐bond) and compositions (such as OH⋅⋅⋅O vs. SH⋅⋅⋅O), while simultaneously providing an estimate to the binding energy between two fragments that can be directly compared with experimental data. Additionally, the LED scheme provides a quantitative framework for discussing cooperative effects between different intermolecular interactions.

As a final remark, it is worth noting that the fp‐LED scheme can be applied to study noncovalent interactions for any system within the range of applicability of the underlying DLPNO‐CCSD(T) scheme, also including those containing transition metals. As an illustrative example, we considered the octahedral [Zn(H_2_O)_6_]^2+^ complex (see the “Zn‐Water” sheet in the SI). We find that the interaction energy between the Zn^2+^ ion and each of the water molecules is −57.4 kcal/mol.

### Breakdown of Binding Energies in Biomolecular Assemblies: Stability of DNA Duplex

**Figure 5:**
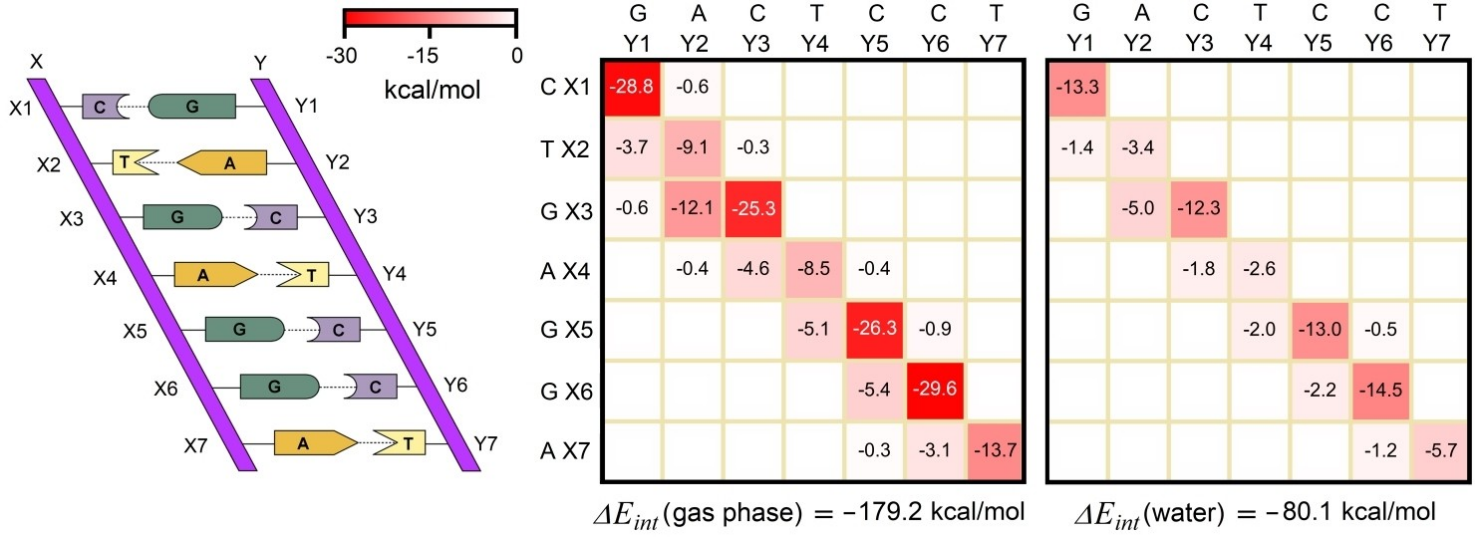
Ladder representation of the section of human DNA considered in this study and fragment‐pairwise LED interaction maps for the decomposition of the inter‐strand binding energy into contributions from pairs of nucleobases calculated at the HFLD/NormalPNO*/def2‐TZVP(−f) level.

Fragment‐pairwise LED can also be used to study the binding energy between complex biomolecular assemblies in terms of the underlying inter‐fragment interactions. As a first prototypical example, we consider the section of human DNA shown in Figure 5, which has been previously examined using the LED scheme in its original formulation.^[57]^ Herein, the binding energy between the two strands is discussed in terms of the individual interactions between the nucleobases, calculated at the HFLD level – which treats all dispersive (inter‐fragment) terms at the DLPNO coupled cluster level and has been shown to reproduce DLPNO‐CCSD(T) results with errors within 1 % for this system^[57]^ (see Supporting Information for details). Note that this structure was constructed by removing the phosphate backbone and saturating the covalent bonds cut with hydrogen atoms, as reported in ref. [57]. The corresponding fp‐LED interaction maps in gas phase and in water are given in Figure 5. The dielectric contribution in implicit solvation scheme was distributed to the pairwise components as described in Section B2 in the SI.

The binding energy between the two strands amounts to −179.2 kcal/mol in the gas phase and −80.1 kcal/mol in water (these figures do not consider the repulsion between the negatively charged backbones, which would make the interaction between the strands highly repulsive in the gas phase, as discussed in the SI). These values primarily originate from interactions between fragment pairs on different strands. The contribution from changes in intra‐strand interactions upon duplex formation is minimal, summing to only 0.71 kcal/mol in the gas phase and 0.16 kcal/mol in water. Consequently, these interactions are not shown in the LED maps in Figure 5.

In both the gas phase and water, similar trends are observed for fragment‐pairwise interactions. Hydrogen‐bond interactions between bases (base pairing) are the largest contributors to the stability of the DNA duplex, as indicated by the diagonal elements in Figure 5. Among these interactions, the G⋅⋅⋅C pairing is much stronger than the A⋅⋅⋅T pairing due to the presence of more hydrogen bonds in G⋅⋅⋅C. On average, the interaction energy is −27.5 kcal/mol for the G⋅⋅⋅C pairing and −10.4 kcal/mol for the A⋅⋅⋅T pairing in the gas phase. For comparison, the best available computational estimates of binding energies for isolated G⋅⋅⋅C and A⋅⋅⋅T pairs are –30/–32 kcal/mol and –15/–17 kcal/mol, respectively, depending on the computational methodology (see Table S3). To a large extent, this difference originates from the fact that the geometry of the pair in the DNA is perturbed compared to its geometric ground state. In solution, the net average effect of base pairing is even smaller, with G⋅⋅⋅C and A⋅⋅⋅T pairs contributing just −13.3 kcal/mol and −3.9 kcal/mol, respectively, to the stability of the DNA duplex.

Although much smaller than base pairing, stacking interactions of the X(n+1)⋅⋅⋅Y(n) type also make a substantial contribution to the duplex stability. In contrast, the effect of X(n)⋅⋅⋅Y(n+1) type stacking is negligible as the corresponding bases are more distant due to the right‐handed helical structure of DNA. We note that the magnitude of the base pairing compared with the stacking interactions aligns well with conventional textbook explanations,^[72],[73]^ and with rupture force measurements obtained via atomic force microscopy (AFM).^[74]^ It is also worth mentioning here that interactions between distant pairs are negligible, thus lending fundamental theoretical support to popular nearest neighbor (NN) models, which assume no interaction between distant bases to predict thermodynamic data of any given DNA sequence.[^75^, ^76^, ^77^, ^78^, ^79^, ^80^, ^81^, ^82^, ^83^] Overall, these results demonstrate that the fragment‐pairwise LED scheme provides a theoretical framework that is uniquely suitable to disentangle the contribution of individual interactions to the stability of complex biomolecular assemblies, with applications across a myriad of fields of chemical research.

### Breakdown of Protein‐Ligand Binding Energies

**Figure 6:**
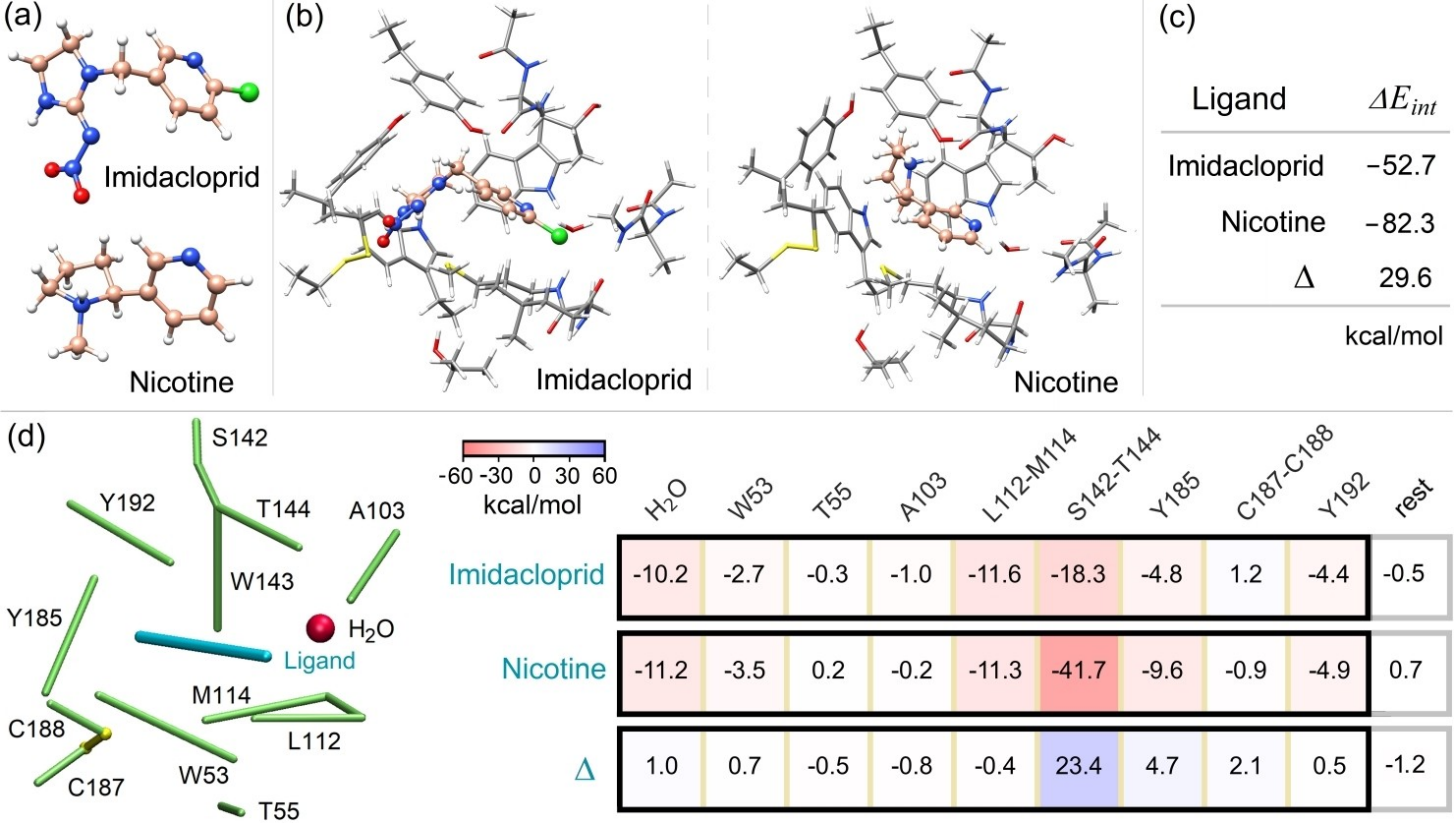
(a) Imidacloprid and nicotine ligands. (b) Binding mode of imidacloprid/nicotine to the “resistant” nAChR protein (see Ref.^[62]^ for the structures). (c) Imidacloprid‐ and nicotine‐nAChR binding energies calculated at the DLPNO‐CCSD(T_0_)/NormalPNO*/def2‐TZVPP level. (d) Breakdown of binding energies into fragment‐pairwise interactions. Only ligand‐residue interactions are explicitly shown. The remaining contributions to the binding energy, representing the change in the strength of residue‐residue interactions upon binding, are comparatively smaller and incorporated into the term labeled “rest”.

Another important application area of the LED scheme is the study of protein‐ligand interactions.^[62]^ As an example, here we consider the binding of two agonists of the nicotinic Acetylcholine Receptor (nAChR), namely imidacloprid and nicotine (Figure 6a).^[62]^ The binding modes of these ligands (Figure 6b) have already been investigated in detail in a number of computational and experimental studies,[^84^, ^85^] including DLPNO‐CCSD(T)/LED investigations.^[62]^ The protein‐ligand binding energies computed at the DLPNO‐CCSD(T) level (see Supporting Information for computational details) are shown in Figure 6c, while the results from the fragment‐pairwise LED scheme are summarized in Figure 6d.

This analysis allows us to quantify the strength of this interaction considering all cooperative effects within the receptor, revealing a significantly stronger binding energy to the protein for nicotine compared to imidacloprid. The interaction between the ligands and the S142/W143/T144 moiety accounts for 79 % of this difference.

### Breakdown of Lattice Energies in Molecular Crystals

The fp‐LED scheme can also be used to disentangle the contribution of individual noncovalent interactions between the monomers to the lattice energies of molecular crystals, providing insights into their stability. This could be used, for example, to understand polymorphism and phase transitions.

**Figure 7:**
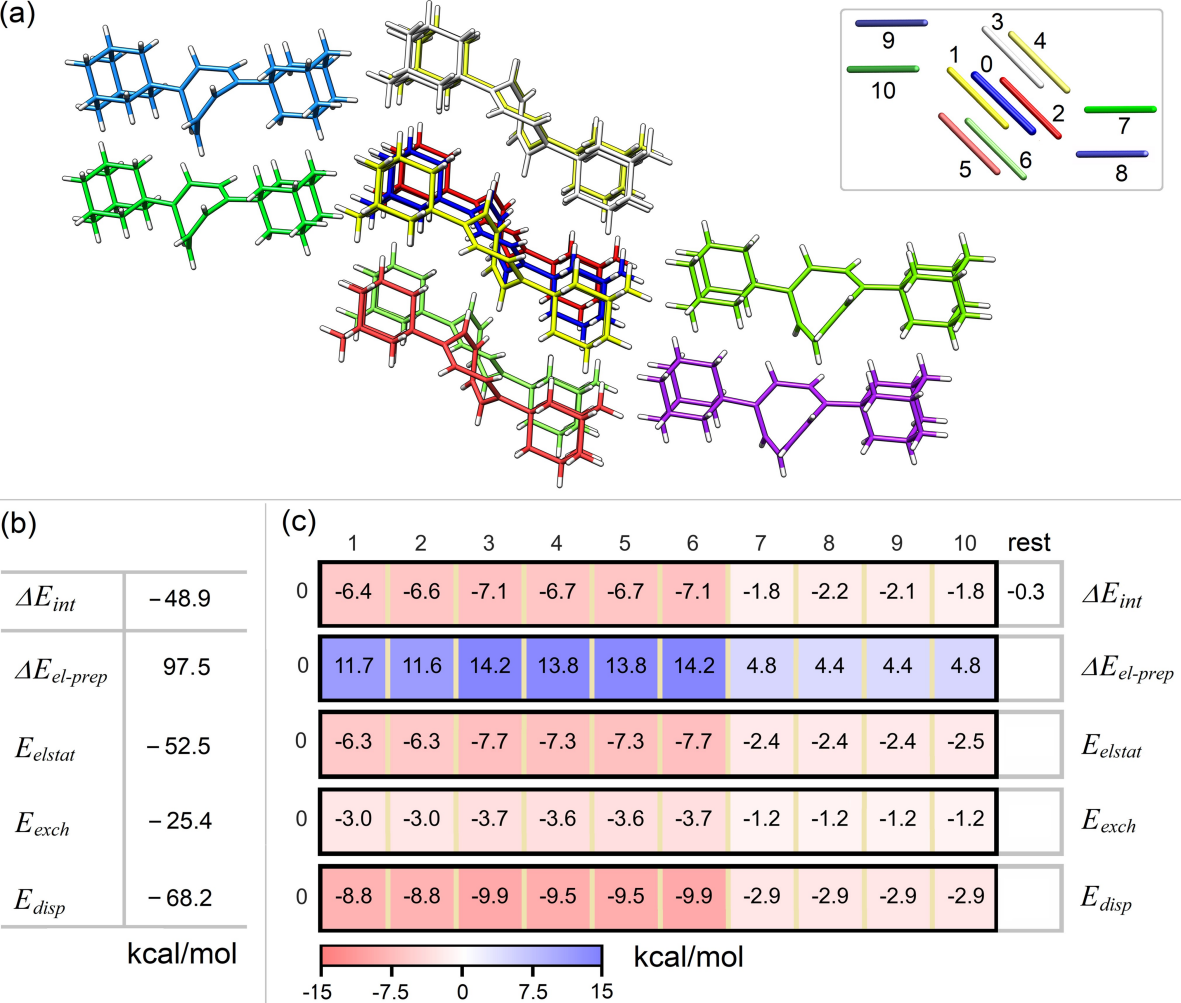
(a) The structure and schematic representation of the 1,4‐di‐adamantyl‐cyclooctatetraene (DIAD‐COT) crystal with monomer labeling (b) LED decomposition of lattice energies ($\Delta E_{int}$ ) into physical contributions calculated at the HFLD/NormalPNO*/def2‐TZVP(−f) level. (c) Breakdown of lattice energies into fragment‐pairwise contributions.

As an example, we consider the lattice energy of the 1,4‐di‐adamantyl‐cyclooctatetraene (DIAD‐COT) crystal^[86]^ at the HFLD level (see Supporting Information for computational details). This was estimated by computing *ΔE_int_* as the energy required to remove the central DIAD‐COT molecule from the molecular cluster shown in Figure 7a, representing a section of the experimentally detected X‐ray crystal structure reported by Schümann et al.^[86]^ Due to symmetry, *ΔE_int_* is twice the electronic component of the lattice energy.^[87]^ Clearly, using a finite cluster rather than a periodic crystal introduces an approximation. However, this error is expected to be minor in crystals composed of nonpolar molecules, such as the DIAD‐COT crystal. In Figure 7b, *ΔE_int_* is decomposed into physical components such as electronic preparation, electrostatics, exchange and dispersion to the interaction using the LED scheme. This decomposition reveals that the major contribution to the stability of this system originates from dispersion forces. In Figure 7c, each of these physical components is decomposed into fragment‐pairwise contributions using the scheme introduced here to assess the effect of each monomer pair on crystal stability.

The pairwise decomposition of the lattice energy reveals two clusters of data: The strongest interaction arises from the parallel alignment of the monomers (**1**–**6**) with the central one **0** while head by head interaction between **0** and **7–10** has much weaker contribution.

## Conclusions

The fragment‐pairwise Local Energy Decomposition methodology, applicable at the DLPNO‐CCSD(T) level, marks a significant step forward in understanding complex interactions within large molecular systems. By quantifying the contributions of individual interactions to binding energies accurately, our approach offers insights that are crucial for various applications in chemical research. Applications spanning molecular clusters, biomolecular assemblies, protein‐ligand interactions, and molecular crystals illustrate the broad applicability and effectiveness of our methodology. We envision that this advancement will catalyze breakthroughs in diverse fields of chemical research, such as materials science, catalysis, and drug discovery.

## Supporting Information

Further details of theory and comparisons with available data in literature (PDF); detailed energetics and heat maps (XLSX).

## Author Contributions

A.A. carried out all the calculations and wrote the original draft. I.F.L. and F.N. contributed to the analysis of the results and to the writing of the manuscript. G.B devised and coordinated the project, and prepared the final version of the manuscript with input from all authors.

## Conflict of Interests

The authors declare no conflict of interest.

1

## Supporting information

As a service to our authors and readers, this journal provides supporting information supplied by the authors. Such materials are peer reviewed and may be re‐organized for online delivery, but are not copy‐edited or typeset. Technical support issues arising from supporting information (other than missing files) should be addressed to the authors.

Supporting Information

Supporting Information

Supporting Information

## Data Availability

The data that support the findings of this study are available from the corresponding author upon reasonable request.
