# Supplementary material for: A Generally Applicable Method for Disentangling the Effect of Individual Noncovalent Interactions on the Binding Energy
Source: Angew Chem Int Ed Engl. 2024 Dec 11;64(12):e202421922. doi: 10.1002/anie.202421922 (PMC11914957; doi:10.1002/anie.202421922)
Supplement: Supplementary file 1 — Supporting Information [file ANIE-64-e202421922-s001.pdf]

Angewandte Chemie
Eine Zeitschrift der Gesellschaft Deutscher Chemiker

Supporting Information

A Generally Applicable Method for Disentangling the Effect of Individual Noncovalent Interactions on the Binding Energy

*A. Altun, I. F. Leach, F. Neese, G. Bistoni**

Supporting Information for

A Generally Applicable Method for Disentangling the Effect of Individual Noncovalent Interactions on the Binding Energy

Ahmet Altun,[a] Isaac F. Leach,[b] Frank Neese,[a] and Giovanni Bistoni[b]*

[a] Max-Planck-Institut für Kohlenforschung, Kaiser-Wilhelm-Platz 1, D-45470 Mülheim an der Ruhr, Germany
[b] Department of Chemistry, Biology and Biotechnology, University of Perugia, 06123 Perugia, Italy
*Email: giovanni.bistoni@unipg.it

Contents

A. Computational Details
B. Theoretical Modeling
B1. Correlation between Electronic Preparation and Inter-fragment Interaction Energy
B2. Fragment-Pairwise Local Energy Decomposition with Implicit Solvation Schemes
B3. Fragment-Pairwise Analysis of Cooperativity Effects
B4. Further Decomposition of Local Energy Decomposition Terms
B5. Comparison of Standard and Fragment Pairwise LED Interaction Energy Maps
C. Comparison of Pairwise Interaction Energies of LED and QTAIM-Based Schemes
D. The Effect of BSSE
E. Comparison of Computed Gas-Phase Interaction Energies of Water Dimer and Watson-Crick Base Pairs
F. Supplementary References

A. Computational Details

All quantum-chemical calculations were carried out with a development version of the ORCA program package based on version 5.0.[S1–S4] The coordinates of the water clusters, the DNA model, the nAChR protein, and the DIAD-COT crystal were taken from refs. [S5], [S6], [S7], and [S8], respectively. Water trimer was optimized at the RI-MP2/aug-cc-pVTZ level. $[Zn(H_2O)6]^{2+}$ complex and dihydroimidazole···BF_3 Lewis pair were optimized at M06-2X-D3(0)/def2-TZVP level (see the corresponding excel sheets of the SI).

In the DLPNO-CCSD(T) calculations of the water clusters, $[Zn(H_2O)6]^{2+}$ complex, dihydroimidazole···BF_3 pair, and Ar_2, “TightPNO” settings[S9,S10] were used and triples contributions were computed by using the iterative (T_1) algorithm.[S11] While aug-cc-pV5Z basis set was employed for Ar_2,.the aug-cc-pVTZ and aug-cc-pVQZ basis sets[S12–S14] were used for the other systems. Their results were then extrapolated to the complete basis set limit, *i.e.*, CBS(3/4),[S15],[S16] and to the complete PNO space limit, *i.e.*, CPS(6/7).[S16],[S17] The interaction energies of $[Zn(H_2O)6]^{2+}$ complex and dihydroimidazole···BF_3 pair were corrected for BSSE. On the prism conformer of water hexamer, we have shown BSSE-corrected and BSSE-uncorrected interaction energies are the same at the CBS limit (see “WaterHexamer-PRISM” excel sheet of the SI).

In the DLPNO-CCSD(T) calculations of the nAChR protein,[S7] NormalPNO* settings[S18] were used and triples contribution was computed by using semi-canonical (T_0) algorithm.[S19] The def2-TZVPP and its matching auxiliary basis sets[S20] were used. HFLD calculations on the DIAD-COT crystal[S8] and DNA duplex[S6] were performed with the NormalPNO* settings.[S18] The def2-TZVP(-f) and its matching auxiliary basis sets[S6] were used. The water environment in the DNA system was treated implicitly by using the Conductor-like Polarizable Continuum Model (CPCM)[S21–S23] within the perturbation theory and energy (PTE) approach.[S24]

LED interaction energy maps were generated from the ORCA output files using the LED Analysis Wizard (LEDAW) tool,[S25] a program suite designed to facilitate the extraction of data from LED outputs of ORCA, enabling the creation of both standard and fragment pair (fp-LED) maps.

B. Theoretical Modeling

B1. Correlation between Electronic Preparation and Inter-fragment Interaction Energy

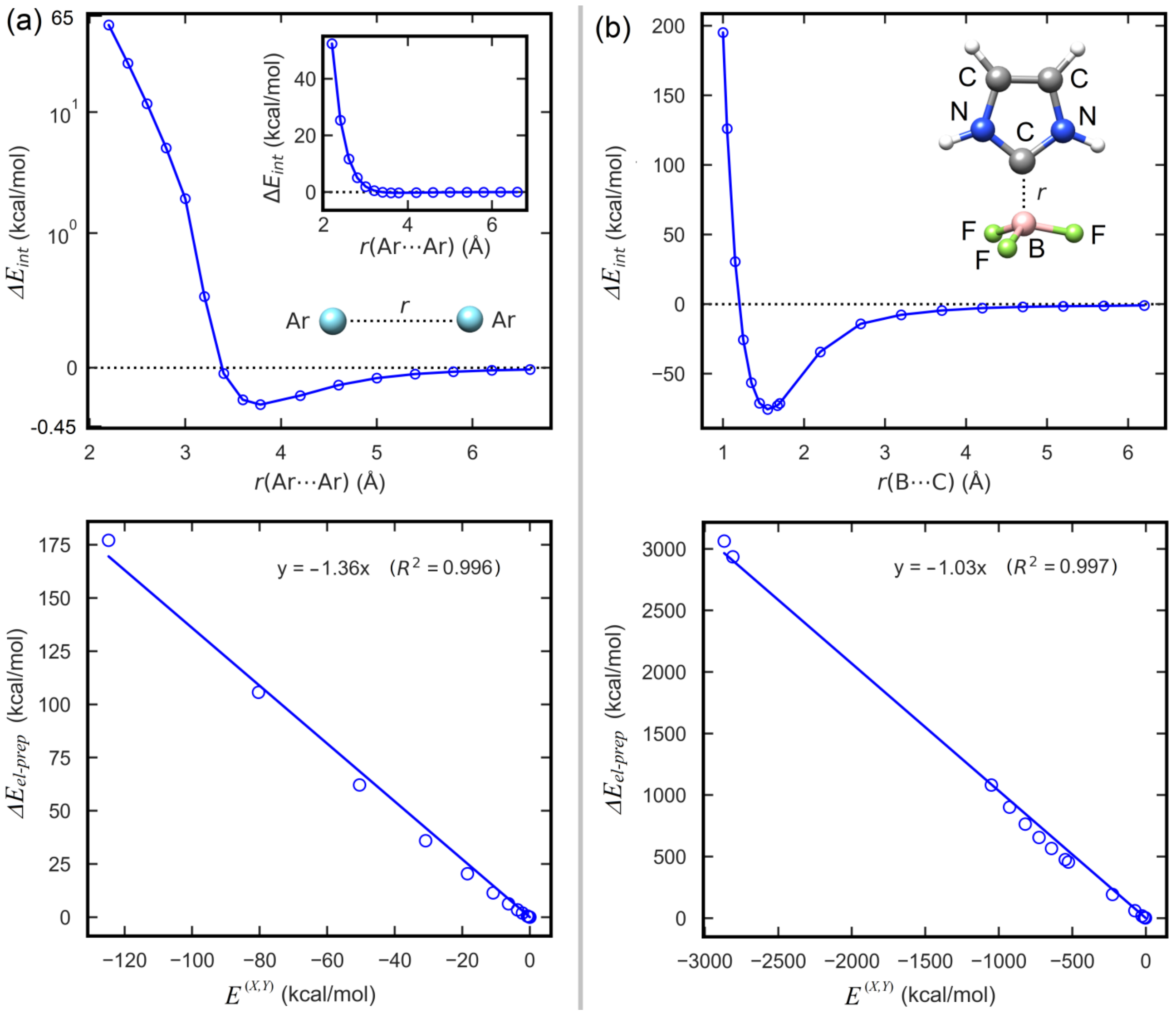

Figure S1. The relaxed potential energy surface (PES, top) and the correlation between the corresponding inter-fragment $E^{(X,Y)}$ and intra-fragment $\Delta E_{el-prep}$ $(\Delta E_X^{el-prep} + \Delta E_Y^{el-prep})$ LED components of the interaction energy (bottom) for (a) Ar···Ar at DLPNO-CCSD(T)/TightPNO/aug-cc-pV5Z level ($T_{CutPairs}$ = 0) (b) dihydroimidazole ($C_3N_2H_4$)···BF_3 at the BSSE-corrected DLPNO-CCSD(T)/CPS(6/7)CBS(3/4) level. For the PES of Ar···Ar, a logarithmic scale is used above y = 1 to make the minimum visible. The plot of the PES with linear y-scale is given as an inset.

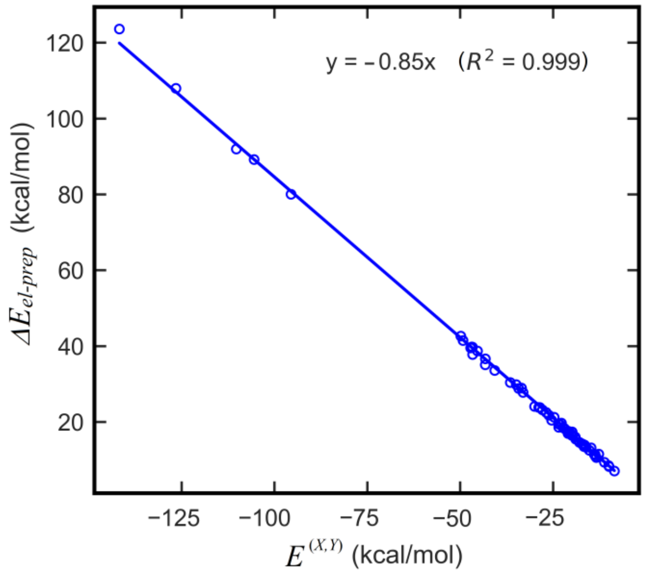

Figure S2. Correlation between inter-fragment $E^{(X,Y)}$ and intra-fragment $\Delta E_{el-prep}$ ($\Delta E_X^{el-prep} + \Delta E_Y^{el-prep}$) LED components of the BSSE-corrected interaction energy for the two-fragment adducts in the S66 benchmark set computed at the DLPNO-CCSD(T)/TightPNO/aug-cc-pVTZ level, where X and Y correspond to each of the monomers in the corresponding adduct.

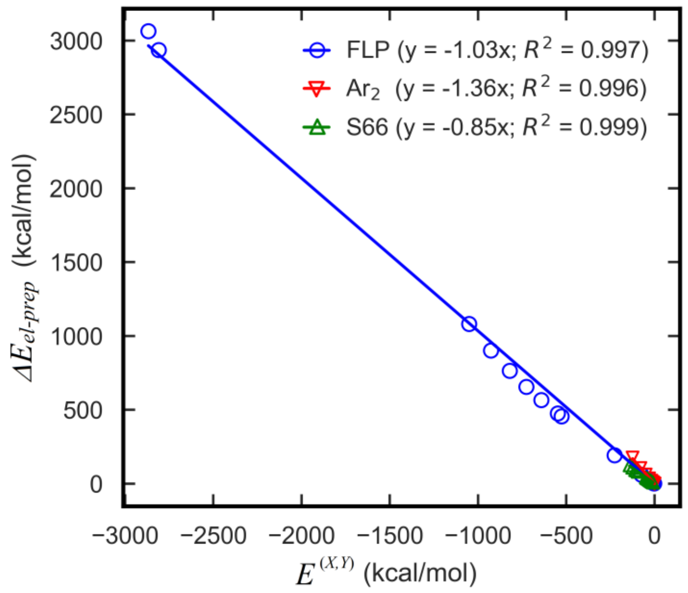

Figure S3. Correlation between inter-fragment $E^{(X,Y)}$ and intra-fragment $\Delta E_{el-prep}$ ($\Delta E_X^{el-prep} + \Delta E_Y^{el-prep}$) LED components of the interaction energy in Ar_2, the S66 benchmark set, and the frustrated Lewis pair (FLP) model $C_3N_2H_4\cdots BF_3$. The data for all systems are shown together in the same figure to make the differences in the energy ranges more apparent, with computational levels as described in the captions of Figures S1 and S2.

As seen in Figures S1-S3, for two-fragment XY systems, the sum of electronic preparation energies ($\Delta E_X^{el-prep} + \Delta E_Y^{el-prep}$) correlates extremely well with $E^{(X,Y)}$ (R^2 = 0.996 for the PES of Ar··Ar; R^2 = 0.997 for the PES of a model frustrated Lewis pair (FLP), i.e., $C_3N_2H_4$·· BF_3; R^2 = 0.999 for S66) in both weak and strong interaction regimes.

B2. Fragment-Pairwise Local Energy Decomposition with Implicit Solvation Schemes

The fp-LED scheme outlined in the main paper is naturally applicable in the case of explicit solvation and can be easily extended to incorporate the effect of the implicit solvent. In this latter case, the electronic energy contains an additional term, namely the dielectric contribution. Hence, the LED decomposed binding energy reads:

$$\Delta E_{int} = \sum_{X>Y} \Delta E_{int,XY} + \Delta E_{diel} \qquad (S1)$$

in which ΔE_{diel} represents the dielectric energy contribution to the interaction energy. The breakdown of this term into fragment-pairwise terms can be done either by treating the solvent charges as an additional fragment, or by distributing the dielectric term among the other fragment-pairwise contributions, as done above for electronic preparation. The latter approach is particularly useful because it results in a number of terms equal to that provided by the decomposition in the gas phase, allowing for a one-to-one comparison between the LED terms in the gas and condensed phases.

This approach was tested on 49 isolated fragment pairs extracted from a seven-nucleobase-long DNA duplex in water (see Figure S4). The results show that ΔE_{diel} correlates very well with $\varepsilon^{(X,Y)}$ (R^2 = 0.988). Hence, the net effect of the implicit solvent is to modulate intermolecular interactions, decreasing their absolute value to an extent that is proportional to their strength. A fragment-pairwise decomposition of the overall ΔE_{diel} into fragment-pairwise terms $\Delta E_{diel}^{(X,Y)}$ can thus be obtained as:

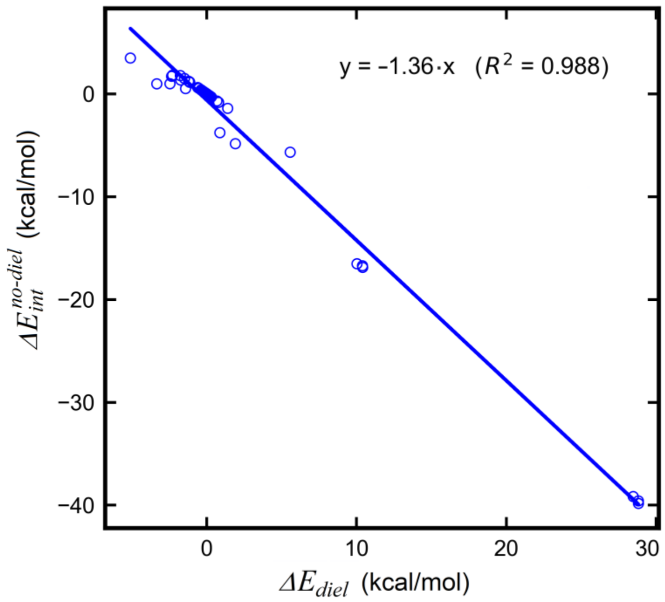

Figure S4. Correlation between dielectric contribution-free interaction energy and dielectric contribution for 49 pairs of nucleobases isolated from a seven-nucleobase-long DNA duplex computed at the HFLD/def2-TZVP(-f) level in implicitly treated water environment.

$$\Delta E_{diel}^{(X,Y)} = \alpha \cdot \varepsilon^{(X,Y)}\ ; \qquad \alpha = \frac{\Delta E_{diel}}{\sum_{X>Y} \varepsilon^{(X,Y)}} \qquad (S2)$$

Note that eq. (S2) ensures that the sum of all $\Delta E_{diel}^{(X,Y)}$ terms equals the non-distributed ΔE_{diel}, *i.e.*,

$$\sum_{X>Y} \Delta E_{diel}^{(X,Y)} = \Delta E_{diel} \qquad (S3)$$

As α is constant for each term in the decomposition, any pairwise term including the dielectric effect is $(1 + \alpha) \cdot \varepsilon^{(X,Y)}$. Hence, this approach is equivalent to the scaling of all the elements of the LED decomposition by the same scalar.[S26]

B3. Fragment-Pairwise Analysis of Cooperativity Effects

Eq. (9) provides a simple breakdown of the electronic energy of a system into fragment-pairwise terms. This scheme also offers a unique opportunity to quantify cooperative effects between different intermolecular interactions. This can be achieved by comparing the terms in eq. (9) with those obtained from the two-body expansion of the binding energy:[S27]

$$\Delta E_{int}^{(2)} = \sum_{X>Y} \Delta E_{int,XY}^{(2)} \qquad (S4)$$

where the superscript (2) is used to indicate the two-body approximation and $\Delta E_{int,XY}^{(2)}$ is the interaction energy of the isolated fragment pair XY , computed by removing all other fragments from the coupled cluster calculation. Accordingly, $\Delta E_{int}^{(2)}$ represents the binding energy of the subsystems in the two-body approximation, *i.e.*, that obtained by neglecting cooperativity of different intermolecular interactions. When eqs. (9) and (S4) are subtracted side by side, the resulting terms represent the effect of higher-body corrections, *i.e.*, the influence of cooperativity into the inter-fragment interactions:

$$\Delta E_{int}^{coop} = \Delta E_{int} - \Delta E_{int}^{(2)} = \left(\sum_{X>Y} \Delta E_{int,XY} - \sum_{X>Y} \Delta E_{int,XY}^{(2)} \right) = \sum_{X>Y} \Delta E_{int,XY}^{coop} \qquad (S5)$$

Eq. (S5) not only allows us to obtain an estimate of the overall cooperativity effect on the binding energy of the subsystems ΔE_{int}^{coop}, but it also provides a quantification for the role of cooperativity on the interaction energy of each fragment pair, $\Delta E_{int,XY}^{coop}$.

It is worth noting that two-body electronic preparation energy of a given XY pair is directly equal to $\Delta E_X^{el-prep,(2)} + \Delta E_Y^{el-prep,(2)}$, and thus no electronic preparation distribution scheme is needed, contrary to the *N*-body case. It is also worth noting that LED of the interaction energy of *N*-fragment systems requires computations only on the *N*-fragment adduct and its constituting fragments. If wanted to assess the effect of the chemical surrounding on the interaction energy of fragment pairs, relatively efficient LED computations on all possible isolated fragment pairs are also necessary.

B4. Further Decomposition of Local Energy Decomposition Terms

Each term in eq. (6) can be readily decomposed into its HF and correlation parts, and then expressed as the sum of pairwise contributions using eq. (9). Hence,

$$\Delta E_{int} = \Delta E_{int}^{HF} + \Delta E_{int}^{C} \tag{S6}$$

where

$$\Delta E_{int}^{HF} = \sum_{X}^{N} \Delta E_{X}^{el-prep,HF} + \sum_{X>Y}^{N} \varepsilon^{(X,Y),HF} = \sum_{X>Y}^{N} \Delta E_{int,XY}^{HF} \tag{S7}$$

$$\Delta E_{int}^{C} = \sum_{X}^{N} \Delta E_{X}^{el-prep,C} + \sum_{X>Y}^{N} \varepsilon^{(X,Y),C} = \sum_{X>Y}^{N} \Delta E_{int,XY}^{C} \tag{S8}$$

There are two possible ways to distribute $\Delta E_{X}^{el-prep}$ and/or its $\Delta E_{X}^{el-prep,HF}$ and $\Delta E_{X}^{el-prep,C}$ components. (*i*) Apply the distribution scheme separately to the HF and correlation energy terms, and then sum the resulting pairwise HF and correlation terms. (*ii*) Apply the distribution scheme directly to the DLPNO-CCSD(T) energy terms. If necessary, apply it also to the HF terms and subtract the distributed DLPNO-CCSD(T) and HF terms to find correlation terms. The pairwise interaction energies and the associated cooperativity components obtained with these two strategies demonstrate negligibly small numerical variations, as shown in Figure S5 on the prism conformer of water hexamer.

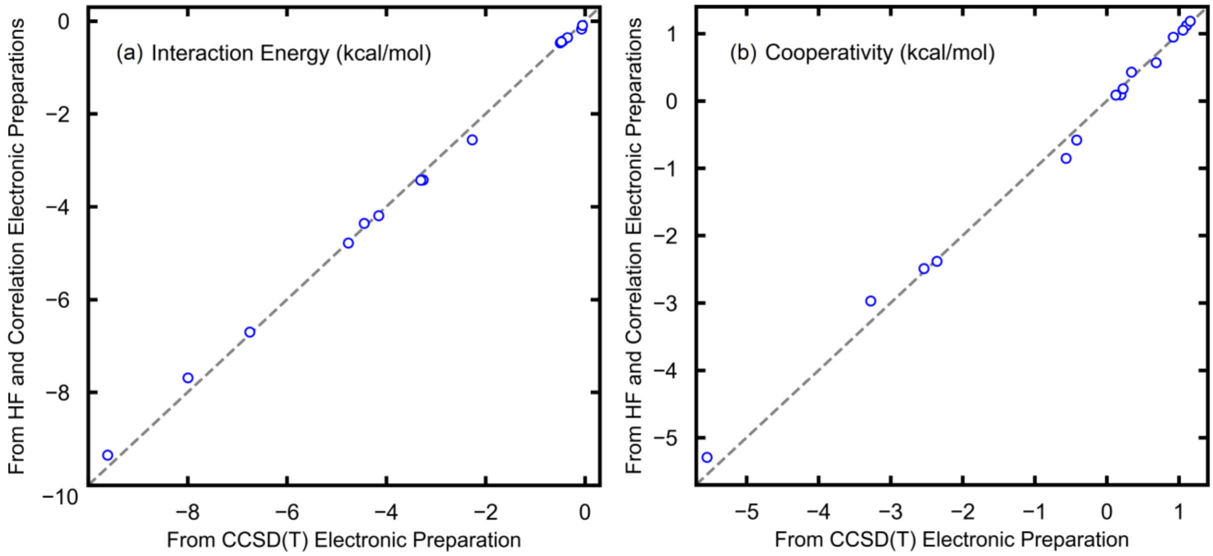

Figure S5. Comparison of the pairwise (a) interaction energy (b) cooperativity LED terms of the prism conformer of water hexamer, obtained distributing DLPNO-CCSD(T)/CPS(6/7)/CBS(3/4) electronic preparation energy directly and distributing individual HF and correlation electronic preparation terms separately. Diagonal dashed line corresponds to the ideal relation.

In this study, we followed the second strategy for DLPNO-CCSD(T). For HFLD, electronic preparation energy was distributed over the HF inter-fragment terms, and the pairwise LD terms were then added to the pairwise distributed HF interaction terms since HFLD incorporates electronic preparation energy only at the HF level.

Each of the pairwise $\varepsilon^{(X,Y),HF}$ and $\varepsilon^{(X,Y),C}$ terms can be further decomposed into electrostatic plus exchange and dispersion plus nondispersive correlation contributions, i.e.,

$$\varepsilon^{(X,Y),HF} = \varepsilon_{elstat}^{(X,Y)} + \varepsilon_{exch}^{(X,Y)} \quad (9)$$

$$\varepsilon^{(X,Y),C} = \varepsilon_{disp}^{(X,Y)} + \varepsilon_{no-disp}^{(X,Y)} \quad (S10)$$

Decomposition of $\varepsilon^{(X,Y),C}$ in eq. (S10) needs further clarification. DLPNO-CCSD(T) correlation is composed of pairs included in the coupled-cluster treatment (strong pairs "SP"), pairs kept at the local MP2 level (weak pairs "WP"), and perturbative triples (T). By assigning pair natural orbitals (PNOs) onto fragments, for each XY fragment pair, inter-fragment part of SP can be decomposed into dispersive ($\varepsilon_{disp}^{(X,Y),C-SP}$) and non-dispersive ($\varepsilon_{no-disp}^{(X,Y),C-SP}$) components. Inter-fragment weak pairs energy is already dispersive in nature, *i.e.*, $\varepsilon^{(X,Y),C-WP} = \varepsilon_{disp}^{(X,Y),C-WP}$. The sum $\varepsilon_{disp,XY}^{C-SP} + \varepsilon_{disp,XY}^{C-WP}$ is the dispersion energy of the pair XY at the CCSD level. HFLD method uses an approximate form of this sum as the dispersion contribution.

As seen in Figure S6, on interacting two-fragment adducts in the S66 benchmark set of NCIs,[S28] strong pairs and triples correlation interaction energies ΔE_{int}^{C-SP} and $\Delta E_{int}^{C-(T),XY}$ as well as their inter-fragment components $E^{(X,Y),C-SP}$ and $E^{(X,Y),C-(T)}$ correlate very well (R^2 = 0.898 and 0.973, respectively) and with almost the same slope. SP dispersion energy $E_{disp}^{(X,Y),C-SP}$ and triples dispersion energy $E_{disp}^{(X,Y),C-(T)}$ can be also expected to correlate with this slope since dispersion energy dominates these two correlation interaction components. Hence, consistent with the suggestion in ref. [S29], these correlations allow obtaining $\varepsilon_{disp}^{(X,Y),C-(T)}$ by scaling $\varepsilon_{disp}^{(X,Y),)Y}$ $interactions\ arising\ from\ s\ mputations.ffect\ and\ dilectric\ term\ of\ \ the\ largest\ atractive\ sitioC-SP$

:

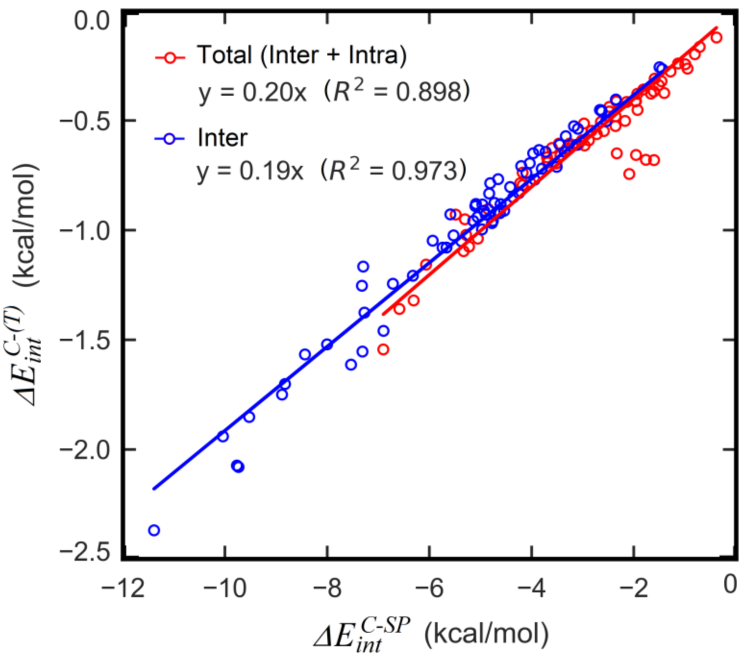

Figure S6. Correlation between BSSE-corrected total strong pairs ΔE_{int}^{C-SP} and total triples $\Delta E_{int}^{C-(T)}$ components of the correlation interaction energy and between their inter-fragment components $E^{(X,Y),C-SP}$ and $E^{(X,Y),C-(T)}$ for the two-fragment adducts in the S66 benchmark set computed at the DLPNO-CCSD(T)/TightPNO/aug-cc-pVTZ level.

$$\varepsilon_{disp}^{(X,Y),C-(T)} = \gamma \cdot \varepsilon_{disp}^{(X,Y),C-SP} \quad ; \quad \gamma = \frac{\varepsilon^{(X,Y),C-(T)}}{\varepsilon^{(X,Y),C-SP}} \tag{S11}$$

The rest of $\varepsilon^{(X,Y),C-(T)}$ is the corresponding inter-fragment non-dispersive triples component $\varepsilon_{no-disp}^{(X,Y),C-(T)}$. Hence, $\varepsilon_{disp}^{(X,Y)}$ and $\varepsilon_{no-disp}^{(X,Y)}$ terms in eq. (S10) are obtained as:

$$\varepsilon_{disp}^{(X,Y)} = \varepsilon_{disp}^{(X,Y),C-SP} + \varepsilon_{disp}^{(X,Y),C-WP} + \varepsilon_{disp}^{(X,Y),C-(T)} \tag{S12}$$

$$\varepsilon_{no-disp}^{(X,Y)} = \varepsilon_{no-disp}^{(X,Y),C-SP} + \varepsilon_{no-disp}^{(X,Y),C-(T)} \tag{S13}$$

B5. Comparison of Standard and Fragment Pairwise LED Interaction Energy Maps

Let us illustrate complete interaction energy map for the interaction of strand *X* (composed of *X1*–*X7*) and strand *Y* (composed of *Y1*–*Y7*) of a seven nucleobase-long DNA duplex in gas phase before and after distributing electronic preparation energy (see Figure S7).

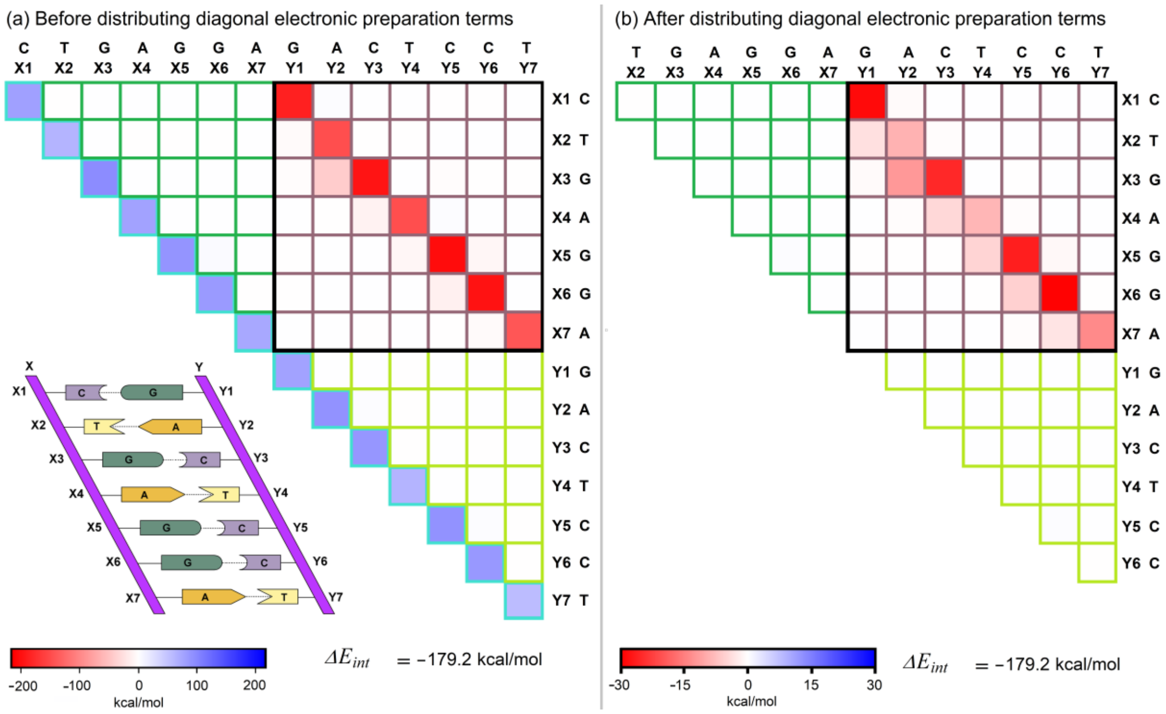

Figure S7. For the interaction of strand X and strand Y of a seven nucleobase-long DNA duplex in gas phase, HFLD/LED interaction energy map of *X1–X7* with *Y1–Y7* (a) before (b) after distributing diagonal electronic preparation energy onto the pairwise HF interaction terms. The cells enclosed by gray lines include also pairwise dispersion terms, which are not considered while distributing diagonal electronic preparation energy terms.

The $\Delta E_X^{el-prep}$ terms in eq. (6) are shown as diagonal elements, enclosed by turquoise lines in Figure S7a. They correspond to the energy associated to the distortion of the electron cloud of each monomer upon adduct formation. The $\varepsilon^{(X,Y)} \equiv \Delta E^{(X,Y)}$ terms in eq. (6) are enclosed in Figure S7a by dark green (see the upper triange) and light green (see the lower triangle), respectively. They correspond to the energy associated to the distortion of the electron cloud of each fragment pair in a given strand by the presence of the other strand, and thus of cooperative in nature. If one of the interacting super systems is composed of only one fragment, as in protein-ligand interactions and lattice energy calculations, such matrix elements do not exist for the corresponding super system. The $\varepsilon^{(X,Y)} \equiv E^{(X,Y)}$ terms in eq. (6) are enclosed by gray lines and correspond to the attractive inter-strand interaction energy terms of fragment pairs when both fragments are located at different strands. Note that the $\varepsilon^{(X,Y)} \equiv \Delta E^{(X,Y)}$ terms are quite small both before and after distributing $\Delta E_X^{el-prep}$ terms. Therefore, we provide in the main paper only the sum of $\Delta E^{(X,Y)}$ terms and label this sum as “rest”. Complete heat maps are provided in the Supporting Information (XLSX).

C. Comparison of Pairwise Interaction Energies of LED and QTAIM-Based Schemes

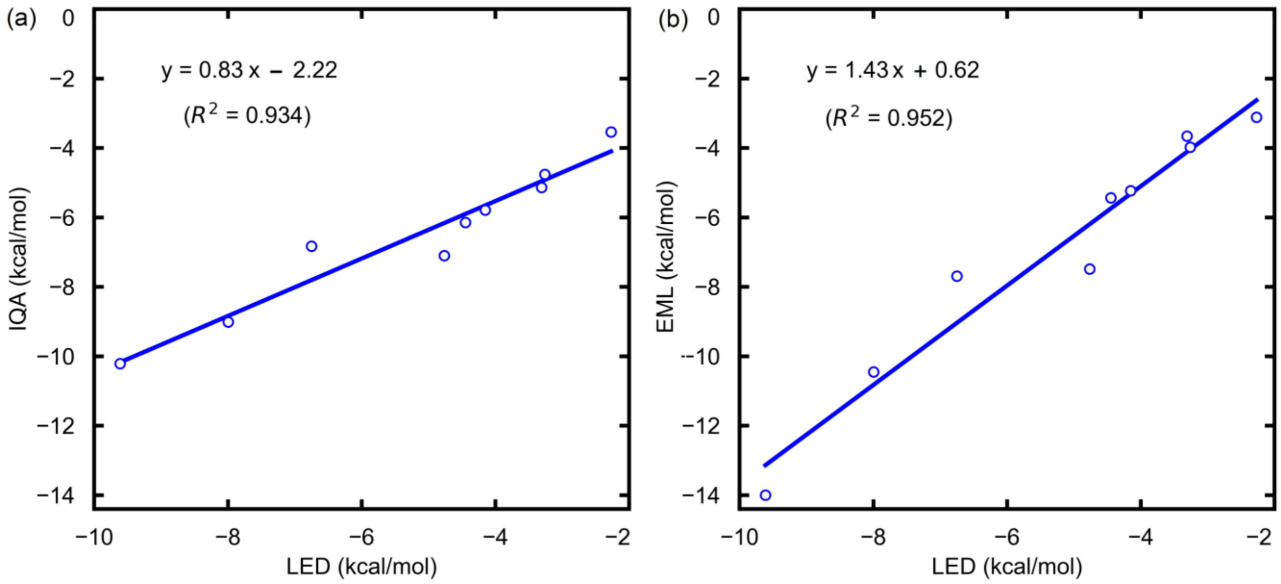

Figure S8. Comparison of pairwise H-bond interaction energies (kcal/mol) for the prism conformer of water hexamer obtained with the LED scheme at the DLPNO-CCSD(T)/CPS(6/7)/CBS(3/4) level and with the QTAIM-based (a) Interacting Quantum Atoms (IQA) and (b) Espinosa-Molins-Lecomte (EML) schemes at the MP2/aug-cc-pVTZ level.[S30]

Pairwise H-bond interaction energies computed with LED and QTAIM-based methods correlate very well.

D. The Effect of BSSE

To obtain BSSE-corrected interaction energies, the monomer energies must be calculated using the basis set of the adduct rather than their respective monomer basis sets. These monomer energies are then subtracted from the corresponding intra-monomer LED component of the adduct energy. Hence, BSSE correction affects only the electronic preparation terms (i.e., diagonal elements on the standard LED maps). However, the terms extrapolated to both CPS and CBS limits are essentially identical, regardless of BSSE correction. As an example, see Sections F and G in "WaterHexamer-PRISM" excel sheet of the SI for the comparison of BSSE-uncorrected and BSSE-corrected LED interaction maps on the prism conformer of the water hexamer, and the corresponding ORCA input files. The deviation of the BSSE-corrected electronic preparation energy per water molecule from the corresponding BSSE-uncorrected energy is presented in Table S1 for this example.

Table S1. The deviation of the BSSE-corrected DLPNO-CCSD(T)/LED electronic preparation energy per water molecule from the corresponding BSSE-uncorrected energy (kcal/mol) in the prism conformer of water hexamer.

	$T_{CutPNO} = 10^{-6}$	$T_{CutPNO} = 10^{-7}$	CPS(6/7)
aug-cc-pVTZ	0.70	0.70	0.70
aug-cc-pVQZ	0.31	0.29	0.28
CBS(3/4)	0.07	0.03	0.01

E. Comparison of Computed Gas-Phase Interaction Energies of Water Dimer and Watson-Crick Base Pairs

Table S2. CCSD(T)/CBS estimate of the interaction energy (kcal/mol) of water dimer and for completeness the corresponding geometrical preparation energy that is not included in the interaction energy

SILVER[a,b]	GOLD[a,b]	HOBZA[a,c]	DLPNO-CCSD(T_1) [a,d]	DLPNO-CCSD(T_1)[e]	$\Delta E_{geo\text{-}prep}$[f]
-4.98	-4.98	-5.01	-4.96	-5.08	0.07

[a] Geometry taken from the S66[S28] benchmark set. [b] Composite explicitly correlated MP2/CCSD(T) result taken from ref. [S31]. [c] Composite MP2/CCSD(T) result taken from ref. [S32]. [d] CPS(6/7)/CBS(3/4)-extrapolated value from ref. [S17]. [e] CPS(6/7)/CBS(3/4)-extrapolated value at the geometry optimized with the same computational settings as water trimer in this work. [f] DLPNO-CCSD(T) value taken from ref. [S33].

Table S3. Computed interaction energies (kcal/mol) of the Watson-Crick (WC) conformers of nucleobase dimers in the gas phase at the HF/CBS, MP2/CBS, MP2/CCSD(T)/CBS, DLPNO-CCSD(T)/CBS, DFT-SAPT/CBS, and HFLD/def2-TZVP(-f) levels and for completeness the corresponding DLPNO-CCSD(T_0)/CBS geometrical preparation energy that is not included in the interaction energy (data collected from various sources, as cited in ref. [S6])

	HF	MP2	MP2/CCSD(T)	DFT-SAPT	DLPNO-CCSD(T_0)	HFLD	$\Delta E_{geo\text{-}prep}$
A···T	-9.9	-16.9	-16.9	-15.7	-16.6	-16.2	1.7
G···C	-24.6	-31.6	-32.1	-30.5	-31.5	-32.8	3.5

F. Supplementary References

[S1] F. Neese, *Wiley Interdiscip. Rev. Comput. Mol. Sci.* **2012**, *2*, 73–78.
[S2] F. Neese, *Wiley Interdiscip. Rev. Comput. Mol. Sci.* **2018**, *8*, e1327.
[S3] F. Neese, F. Wennmohs, U. Becker, C. Riplinger, *J. Chem. Phys.* **2020**, *152*, 224108.
[S4] F. Neese, *Wiley Interdiscip. Rev. Comput. Mol. Sci.* **2022**, *12*, e1606.
[S5] M. Alkan, P. Xu, M. S. Gordon, *J. Phys. Chem. A* **2019**, *123*, 8406–8416.
[S6] A. Altun, M. Garcia-Ratés, F. Neese, G. Bistoni, *Chem. Sci.* **2021**, *12*, 12785–12793.
[S7] M. E. Beck, C. Riplinger, F. Neese, G. Bistoni, *J. Comput. Chem.* **2021**, *42*, 293–302.
[S8] J. Schümann, L. Ochmann, J. Becker, A. Altun, I. Harden, G. Bistoni, P. Schreiner, *J. Am. Chem. Soc.* **2023**, *145*, 2093–2097.
[S9] C. Riplinger, P. Pinski, U. Becker, E. F. Valeev, F. Neese, *J. Chem. Phys.* **2016**, *144*, 024109.
[S10] D. G. Liakos, M. Sparta, M. K. Kesharwani, J. M. L. Martin, F. Neese, *J. Chem. Theory Comput.* **2015**, *11*, 1525–1539.
[S11] Y. Guo, C. Riplinger, U. Becker, D. G. Liakos, Y. Minenkov, L. Cavallo, F. Neese, *J. Chem. Phys.* **2018**, *148*, 011101.
[S12] T. H. Dunning, *J. Chem. Phys.* **1989**, *90*, 1007–1023.
[S13] N. B. Balabanov, K. A. Peterson, *J. Chem. Phys.* **2005**, *123*, 064107.
[S14] K. A. Peterson, T. H. Dunning, *J. Chem. Phys.* **2002**, *117*, 10548–10560.
[S15] F. Neese, A. Hansen, D. G. Liakos, *J. Chem. Phys.* **2009**, *131*, 064103.
[S16] A. Altun, S. Ghosh, C. Riplinger, F. Neese, G. Bistoni, *J. Phys. Chem. A* **2021**, *125*, 9932–9939.
[S17] A. Altun, F. Neese, G. Bistoni, *J. Chem. Theory Comput.* **2020**, *16*, 6142–6149.
[S18] A. Altun, F. Neese, G. Bistoni, *J. Chem. Theory Comput.* **2019**, *15*, 5894–5907.
[S19] C. Riplinger, B. Sandhoefer, A. Hansen, F. Neese, *J. Chem. Phys.* **2013**, *139*, 134101.
[S20] A. Schäfer, C. Huber, R. Ahlrichs, *J. Chem. Phys.* **1994**, *100*, 5829.
[S21] V. Barone, M. Cossi, J. Tomasi, *J. Chem. Phys.* **1998**, *107*, 3210.
[S22] V. Barone, M. Cossi, *J. Phys. Chem. A* **1998**, *102*, 1995–2001.
[S23] M. Cossi, N. Rega, G. Scalmani, V. Barone, *J. Comput. Chem.* **2003**, *24*, 669–681.

[S24] R. Cammi, *J. Chem. Phys.* **2009**, *131*, 164104.
[S25] A. Altun, LEDAW - LED Analysis Wizard for Automating Local Energy Decomposition Analysis Using ORCA Outputs: A Python-Based Program Package, https://github.com/ahmetaltunfatih/LEDAW, 2024.
[S26] G. Park, E. C. Wralstad, N. Faginas-Lago, K. Qian, R. T. Raines, G. Bistoni, C. C. Cummins, *ACS Cent. Sci.* **2024**, *10*, 1415–1422.
[S27] S. Ghosh, F. Neese, R. Izsák, G. Bistoni, *J. Chem. Theory Comput.* **2021**, *17*, 3348–3359.
[S28] J. Řezáč, K. E. Riley, P. Hobza, *J. Chem. Theory Comput.* **2011**, *7*, 2427–2438.
[S29] Q. Lu, F. Neese, G. Bistoni, *Phys. Chem. Chem. Phys.* **2019**, *2019*, 11569–11577.
[S30] J. M. Guevara-Vela, E. Romero-Montalvo, V. A. Mora Gómez, R. Chávez-Calvillo, M. García-Revilla, E. Francisco, Á. M. Pendás, T. Rocha-Rinza, *Phys. Chem. Chem. Phys.* **2016**, *18*, 19557–19566.
[S31] M. B. Ahirwar, S. R. Gadre, M. M. Deshmukh, *J. Phys. Chem. A* **2020**, *124*, 6699–6706.
[S32] J. Řezáč, P. Hobza, *Chem. Rev.* **2016**, *116*, 5038–5071.
[S33] A. Altun, F. Neese, G. Bistoni, *Beilstein J. Org. Chem.* **2018**, *14*, 919–929.
